# O-GlcNAc Transferase Regulates Angiogenesis in Idiopathic Pulmonary Arterial Hypertension

**DOI:** 10.3390/ijms20246299

**Published:** 2019-12-13

**Authors:** Jarrod W. Barnes, Liping Tian, Stefanie Krick, E. Scott Helton, Rebecca S. Denson, Suzy A. A. Comhair, Raed A. Dweik

**Affiliations:** 1 Division of Pulmonary, Allergy and Critical Care Medicine, Department of Medicine, The University of Alabama at Birmingham, THT 422, 1720 2nd Ave S, Birmingham, AL 35294-0006, USA; skrick@uab.edu (S.K.); esh@uab.edu (E.S.H.);; 2 Department of Inflammation & Immunity, Cleveland Clinic, 9500 Euclid Ave, Cleveland, OH 44195, USA; tianl@ccf.org (L.T.); comhais@ccf.org (S.A.A.C.); dweikr@ccf.org (R.A.D.); 3 Respiratory Institute, Cleveland Clinic, 9500 Euclid Ave, Cleveland, OH 44195, USA

**Keywords:** O-GlcNAc, IPAH, angiogenesis, OGT, VEGF

## Abstract

Idiopathic pulmonary arterial hypertension (IPAH) is considered a vasculopathy characterized by elevated pulmonary vascular resistance due to vasoconstriction and/or lung remodeling such as plexiform lesions, the hallmark of the PAH, as well as cell proliferation and vascular and angiogenic dysfunction. The serine/threonine hydroxyl-linked N-Acetylglucosamine (O-GlcNAc) transferase (OGT) has been shown to drive pulmonary arterial smooth muscle cell (PASMC) proliferation in IPAH. OGT is a cellular nutrient sensor that is essential in maintaining proper cell function through the regulation of cell signaling, proliferation, and metabolism. The aim of this study was to determine the role of OGT and O-GlcNAc in vascular and angiogenic dysfunction in IPAH. Primary isolated human control and IPAH patient PASMCs and pulmonary arterial endothelial cells (PAECs) were grown in the presence or absence of OGT inhibitors and subjected to biochemical assessments in monolayer cultures and tube formation assays, in vitro vascular sprouting 3D spheroid co-culture models, and de novo vascularization models in NODSCID mice. We showed that knockdown of OGT resulted in reduced vascular endothelial growth factor (VEGF) expression in IPAH primary isolated vascular cells. In addition, specificity protein 1 (SP1), a known stimulator of VEGF expression, was shown to have higher O-GlcNAc levels in IPAH compared to control at physiological (5 mM) and high (25 mM) glucose concentrations, and knockdown resulted in decreased VEGF protein levels. Furthermore, human IPAH PAECs demonstrated a significantly higher degree of capillary tube-like structures and increased length compared to control PAECs. Addition of an OGT inhibitor, OSMI-1, significantly reduced the number of tube-like structures and tube length similar to control levels. Assessment of vascular sprouting from an in vitro 3D spheroid co-culture model using IPAH and control PAEC/PASMCs and an in vivo vascularization model using control and PAEC-embedded collagen implants demonstrated higher vascularization in IPAH compared to control. Blocking OGT activity in these experiments, however, altered the vascular sprouting and de novo vascularization in IPAH similar to control levels when compared to controls. Our findings in this report are the first to describe a role for the OGT/O-GlcNAc axis in modulating VEGF expression and vascularization in IPAH. These findings provide greater insight into the potential role that altered glucose uptake and metabolism may have on the angiogenic process and the development of plexiform lesions. Therefore, we believe that the OGT/O-GlcNAc axis may be a potential therapeutic target for treating the angiogenic dysregulation that is present in IPAH.

## 1. Introduction

Pulmonary arterial hypertension (PAH) is a syndrome comprised of overlapping complications with varying origins that presents with many phenotypes [1,2,3]. The idiopathic form of pulmonary arterial hypertension (IPAH) is progressive and results in the deterioration of cardiopulmonary function and premature death [4,5]. Presently, IPAH is considered a vasculopathy characterized by elevated pulmonary vascular resistance due to vasoconstriction and/or lung remodeling such as plexiform lesions, the hallmark of the PAH, as well as cell proliferation and vascular and angiogenic dysfunction [4,5,6,7,8,9,10,11,12]. On a molecular level, increased expression of the vascular endothelial growth factor (VEGF) ligand and VEGF receptor (VEGFR), both instrumental for de novo vascularization, have been observed in the plexogenic lesions [13,14,15]. These findings suggest that the cellular makeup of the plexogenic lesions is endothelial in nature; however, other cell types cannot be ruled out since they also express VEGF/VEGFR [16,17].

Metabolic dysregulation has emerged as a major area of research in the pathobiology of IPAH [7,18,19,20,21,22]. In particular, altered metabolic functions, including increased glucose uptake/metabolism, have been characterized in IPAH, including higher glycolytic rates than healthy individuals [19,22,23,24,25,26,27,28]. Altered glucose metabolic flux into cells may result in glucose intolerance in IPAH patients. We recently described a potential consequence of increased glucose uptake and shunt in the hexosamine biosynthetic pathway (HBP), a known metabolic regulator and a sensor of nutrient flux [28]. Our findings demonstrated that altered glucose uptake and HBP shunt contributed to the increased O-linked N-Acetylglucosamine (O-GlcNAc) transferase expression and activity, which was associated with increased O-GlcNAc levels in pulmonary arterial smooth muscle cell and endothelial cells (PASMCs and PAECs, respectively), PASMC proliferation, and IPAH clinical worsening.

HBP flux regulates the biosynthesis of the sugar nucleotide uridine diphosphate (UDP)-GlcNAc, which serves as a building block for glycan processing, including O-GlcNAc [29,30]. For the O-GlcNAc modification of proteins, the ‘GlcNAc’ moiety from UDP-GlcNAc is transferred and covalently attached to serine and threonine residues via OGT [31,32]. O-GlcNAc is abundant in the cell, including the cytoplasm, nucleus, and mitochondria, and is widely recognized for its antagonistic role to other post-translational modifications such as phosphorylation [32,33,34]. Similar to protein phosphorylation, the addition or removal of O-GlcNAc by O-GlcNAc hydrolase (OGA) is a dynamic process under the influence of the cellular environment (i.e., stress, hormones, and nutrient flux). Alterations in glucose uptake can modulate protein O-GlcNAc levels and may be a driving force for regulating protein activity and function. The O-GlcNAc modification of proteins in vascular disease and angiogenesis has been well studied [35,36,37,38,39,40,41]. However, the role of OGT/O-GlcNAc in IPAH is still poorly understood. Several IPAH dysregulated processes other than our previously published findings on cell proliferation [28], such as vasoconstriction and increased angiogenesis, may be impacted by altered glucose uptake and increased OGT/O-GlcNAc levels. In this report, we demonstrate that increased OGT/O-GlcNAc levels contribute to altered vascular sprouting and impaired de novo vascularization in IPAH. These findings suggest a role for increased glucose uptake and modulation of O-GlcNAc levels on specificity protein 1 (SP1), which is known to regulate VEGF ligand.

## 2. Results

### 2.1. The OGT/O-GlcNAc Axis Regulates VEGF-A Expression by the O-GlcNAc Modification of SP-1

**Figure 1:**
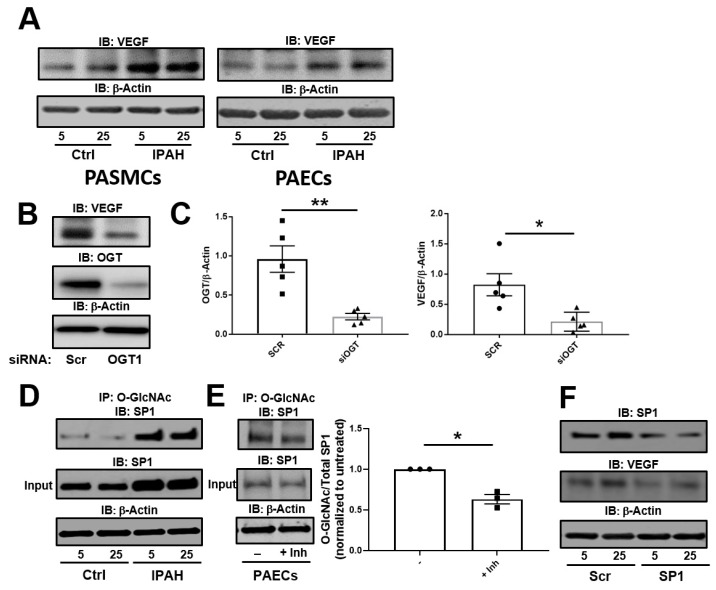
The OGT/O-GlcNAc axis regulates VEGF expression by the O-GlcNAc modification of SP-1. (**A**) Representative Immunoblots of VEGF and β-Actin from IPAH PASMCs and PAECs following 5 or 25 mM glucose administration as described in the Methods section. (**B**) PAECs were transfected with scramble or OGT siRNA. OGT and VEGF expression was determined by Immunoblot analysis and quantitated (**C**). Immunoprecipitation was performed using 1 g of a PAEC lysate following different glucose concentrations as described in the methods (**D**) or IP in the presence or absence of OGT inhibitor (Inh) (**E**). The samples were incubated with O-GlcNAc antibody followed by addition of protein L beads, SDS-PAGE, and Western blot analysis. (**F**) A representative Western blot of SP1 and VEGF following SP1 gene silencing using siRNA in the presence of different glucose concentrations. Error bars indicate standard error of the mean (SEM). * *p* < 0.05 and ** *p* < 0.01.

We previously showed that increased glucose uptake facilitates pathways involved in the augmentation of glycosylation and cell proliferation [28] as well as cell migration [26] in IPAH PASMCs. To determine the role of dysregulated cellular glucose uptake on VEGF-A expression in these cells, we analyzed VEGF-A by Western blot under physiological and high glucose concentrations. As shown in Figure 1A (left panel), VEGF-A expression in IPAH PASMCs was increased in both 5 and 25 mM glucose concentrations at 24 h compared to control PASMCs. Furthermore, similar results were demonstrated in PAECs known to express VEGF-A ligand (Figure 1A, right panel). Silencing of the glucose responsive enzyme, OGT, in PAECs resulted in reduced VEGF-A protein expression (Figure 1B,C). These results suggest that VEGF-A expression is linked to alterations in glucose uptake and OGT activity, which is consistent with prior reports [42].

VEGF-A expression has been shown to be augmented by OGT activation of SP1 through the O-GlcNAc modification [42]. To determine the difference in levels of O-GlcNAc on SP-1, we Immunoprecipitated (IP) O-GlcNAc modified proteins from PAECs and Immunoblotted for SP1 in low and high glucose concentrations. As shown in Figure 1D, the O-GlcNAc modification on SP1 was increased in the IPAH PAECs for both high and low glucose concentrations compared to control cells as determined by IP. Additionally, levels of O-GlcNAc modification were reduced on SP1 in PAECs following a 24 h incubation with the OGT inhibitor, OSMI-1 (25 µM) (Figure 1E). Upon knockdown of SP1 in IPAH PAECs, VEGF-A expression was reduced compared to the non-target (scramble) siRNA control (Figure 1F). Altogether, these findings suggest that SP1 has higher protein O-GlcNAc levels in IPAH compared to controls and loss SP1 expression results in a reduction of VEGF-A ligand.

### 2.2. OGT Regulates Vascular Endothelial Tube Formation and Vascular Sprouting in IPAH

**Figure 2:**
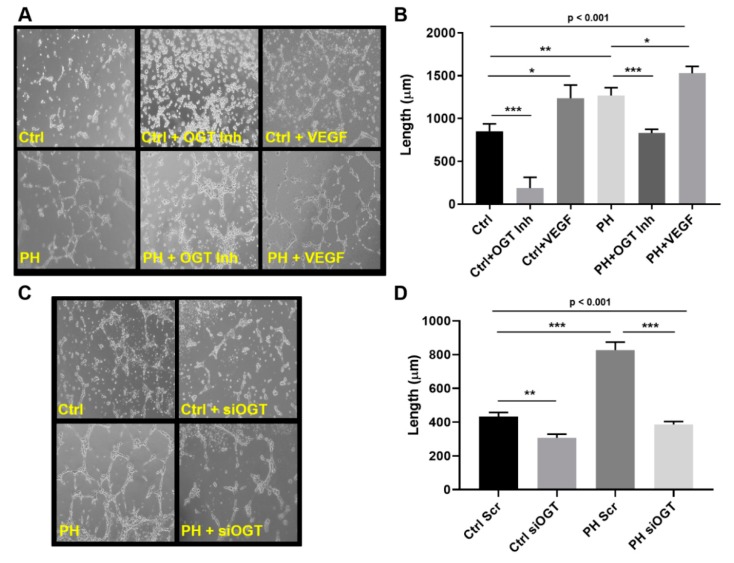
OGT regulates vascular endothelial tube formation in IPAH PAECs. Tube formation from control (*n* = 3) and IPAH (*n* = 3) human PAECs at 6 h following treatment with OGT inhibitor and VEGF ligand (**A**,**B**) or OGT knockdown with siRNA (**C**,**D**). Tube formation was measured using snapshots obtained from an Olympus CKX31 microscope calibrated at the 10X objective (**B**,**D**). A minimum of 12 fields were taken for each condition and tube length was calculated using Image-Pro^®^ Plus 7.0. Ctrl = Control; PH = Idiopathic pulmonary arterial hypertension, OGT Inh. = O-GlcNAc Transferase Inhibitor; VEGF = Vascular Endothelial Growth Factor, and siOGT = OGT siRNA knockdown. Error bars indicate standard error of the mean (SEM). * *p* < 0.05; ** *p* < 0.01; and *** *p* < 0.001.

A common feature of IPAH is new angiogenic growth, a feature that can be regulated by changes in glucose utilization [35,43]. To determine the role that dysregulated glucose metabolism and the OGT/O-GlcNAc axis have in IPAH vascular sprouting, we assessed control and IPAH primary pulmonary arterial endothelial cells (PAECs) for tube formation at 6 h following OGT inhibition. As shown in Figure 2A, IPAH PAECs form more capillary-like tube structures and increased tube length compared to the control PAECs (Figure 2B, PH: 1269.0 ± 91.1 vs. Ctrl: 851.9 ± 86.4, *p* < 0.01). As a control, VEGF ligand was administered in parallel experiment and showed an increase in overall tube formation in both control and IPAH PAECs (Figure 2A,B, Ctrl: 851.9 ± 86.4 vs. Ctrl + VEGF: 1238.0 ± 152.8, *p* < 0.05 and PH: 1269.0 ± 91.1 vs. PH + VEGF: 1530 ± 80.2, *p* < 0.05). Conversely, blocking OGT activity with the OGT inhibitor, OSMI-1 (25 µM), resulted in a decrease in both tube formation and length in control and IPAH PAECs (Figure 2A,B, Ctrl: 851.9 ± 86.4 vs. Ctrl + OGT Inh: 187.9 ± 125.5, *p* < 0.001 and PH: 1269.0 ± 91.1 vs. PH + OGT Inh: 831.3 ± 42.4, *p* < 0.001). Similar findings were demonstrated with knockdown of OGT in the endothelial cells using siRNA (Figure 2C,D, Ctrl Scr: 433.5 ± 23.4 vs. Ctrl siOGT: 306.6 ± 22.1, *p* < 0.01 and PH Scr: 827.4 ± 47.09 vs. PH siOGT: 386.6 ± 17.4, *p* < 0.001).

**Figure 3:**
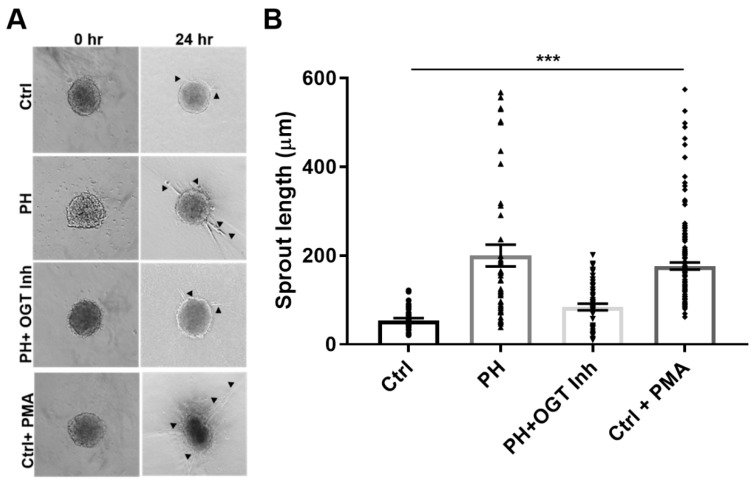
OGT regulates vascular sprouting in a 3D co-culture model containing IPAH PAECs and PASMCs. (**A**) Representative spheroids from control and IPAH human PAECs:PASMCs (4:1) with treatments and times indicated. (**B**) Box and whiskers plots of the sprouting length. Vascular sprouting was measured using snapshots obtained from an Olympus CKX31 microscope calibrated at the 10X objective. A minimum of 20 fields were used for the sprouting analysis. Arrow indicate multiple sprouts. Abbrv. Ctrl = Control; PH = Idiopathic pulmonary arterial hypertension; OGT Inh. = O-GlcNAc Transferase Inhibitor; and PMA = phorbol-12 myristate 13-acetate. Error bars indicate standard error of the mean (SEM). ANOVA was used for multiple comparisons and *** denotes *p* < 0.001.

In a 3-D model of vascular sprouting, IPAH SMC:EC spheroids embedded in collagen had increased vascular sprouting and length at 24 h compared to controls (Figure 3, PH: 200.2 ± 24.6 vs. Ctrl: 54.2 ± 4.9, *p* < 0.001). The increased vascular sprouting was consistent with phorbol-12-myristate-13 acetate (PMA) treatment on control spheroids (Figure 3, PH: 200.2 ± 24.6 vs. Ctrl + PMA: 176.8 ± 7.8, *p* > 0.05), which has been shown to increase cellular glucose uptake [44,45]. Conversely, OGT inhibition with OSMI-1 in the IPAH SMC:EC 2D spheroids demonstrated reduced sprouts and sprout length, similar to controls (PH + OGT Inh: 84.1 ± 7.5 vs. Ctrl: 54.2 ± 4.9, *p* > 0.05) and opposite of IPAH (PH: 200.2 ± 24.6 vs. PH + OGT Inh: 84.1 ± 7.5, *p* < 0.001). These data combined suggest that IPAH vascular cells have increased sprouting consistent with PMA-treated (spontaneous glucose uptake) control spheroids and OGT inhibition can reduce the angiogenic potential and vascular sprouting in IPAH vascular cells.

### 2.3. OGT Inhibition Attenuates De Novo Vascularization in a Humanized Angiogenic Mouse Model

**Figure 4:**
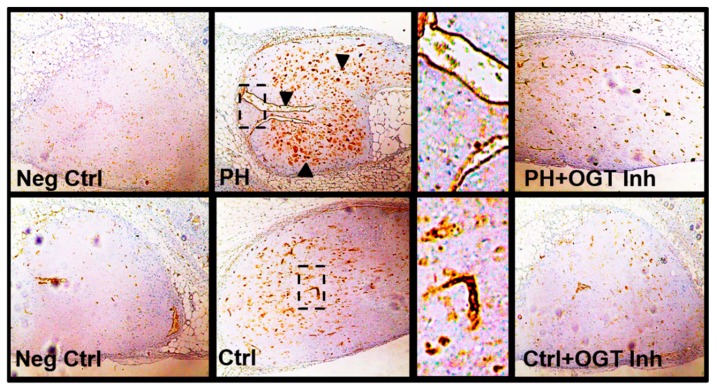
OGT inhibition attenuates de novo vascularization in a humanized in vivo mouse model. Representative Immunohistochemical stains showing increased de novo vasculogenesis of IPAH PAECs compared to control PAECs. Collagen-embedded PAECs were surgically implanted into the abdominal wall, between the dermis and peritoneum of humanized NODSCID mice as described (4,5). The collagen discs were removed, fixed, paraffin embedded, and stained with anti-human CD31 (only detects human epitope). Experiments were done with and without OGT inhibitor (OSMI-1) to assess the regulation of OGT on vasculogenesis. Arrowheads indicate areas of intense CD31 positive staining. Images were obtained from an Olympus CKX31 microscope using a 10X objective. Dotted box denotes a zoomed in image (to the right of image). Arrowheads denote areas of dense CD31 positive staining. Abbrv. Neg Ctrl = Negative Control (staining without primary antibody); PH = Idiopathic pulmonary arterial hypertension; and OGT Inh. = O-GlcNAc Transferase Inhibitor (OSMI-1).

To determine the ability of OGT to regulate vascularization in vivo, a model for de novo vasculogenesis was performed. This technique allows the study of vasculogenic capacity of endothelial colony forming cells within collagen-embedded endothelial cell cultures surgically implanted into the abdominal wall, between the dermis and peritoneum of humanized NODSCID mice [46,47]. The collagen-embedded IPAH PAECs showed increased de novo vascularization compared to control implants as determined by anti-human CD31 positive staining [recognizes human only [46,48,49]; clone JC70/A, DAKO, USA) within the implant (Figure 4). Upon addition of the OSMI-1 into the collagen embedded IPAH or control PAECs, de novo vascularization within the collagen implant was reduced (Figure 4). Altogether, these data suggest that OGT is critical in the regulation of de novo vascularization in IPAH PAECs.

## 3. Discussion

In this report, we show that both IPAH smooth muscle cells and endothelial cells have increased VEGF protein expression even at high glucose. Upon silencing of OGT or SP1 expression, VEGF protein levels are reduced. In addition, IPAH PAECs have higher O-GlcNAc modified SP1, which has been shown to transcriptionally regulate VEGF expression [42]. Furthermore, inhibition of OGT in an endothelial tube formation assay reduced tube length and formation. Combining PAECs and PASMCs in a 3D vascular sprouting model, IPAH vascular spheroids spontaneously produced more vascular sprouts and had increased sprout length compared to the control PAEC:PASMC spheroids. This finding was consistent with PMA treatment of control vascular spheroids, which has been shown to increase glucose uptake through modulation of protein kinase C (PKC) [45]. In line with this, blocking OGT activity reduced de novo vasculogenesis in PAEC-embedded collagen implants within the dermis and peritoneum of humanized NODSCID mice. Altogether, these data, combined with our previous studies [26,28], suggest that increased OGT activity, through SP1/VEGF, may contribute to the vascular dysregulation in IPAH.

Pulmonary arterial hypertension has been characterized as a vasculopathy and the hallmark of PAH is the formation of plexiform lesions [7,8,11]. These complex vascular formations originate from the remodeled pulmonary arterial lumen and small precapillary arteries and contain endothelial as well as smooth muscle cells that express many angiogenic factors [7,48]. Furthermore, the molecular signals responsible for angiogenesis within the plexiform lesions have been thought to be a continuous process involving the formation of new blood vessels from pre-existing blood vessels driven by their dysregulated molecular make-up (e.g., altered VEGF signaling) called “misguided angiogenesis,” a term given by Tuder and Voelkel [13,15,49,50,51,52] owever, the development and overall significance of the plexiform lesion are not yet fully understood. It has been generally thought that the angiogenic processes that coordinate the vascular remodeling and dysfunction in PAH are solely determined by genetic cues, but many recent reports have shown that dysregulated metabolism may be involved [51,52,53]. Our previous report showed that slight changes in OGT levels can result in drastic changes to cell proliferation and/or metabolism [28]. In the IPAH PASMCs, where OGT is increased due to basal metabolic changes associated with these cells, a reduction of OGT levels resulted in decreased proliferation to normal proliferation similar to controls. Interestingly, overexpression of OGT or knockdown of OGT in control cells can result in cell death or a complete reduction in cell proliferation, respectively (see Supplemental Figure S1). This suggests that (in certain cell types) vast changes in OGT expression may alter a homeostatic threshold. For example, too much or too little OGT activity can cause robust effects resulting in regulated cell death or increased proliferation; therefore, the OGT levels must be maintained. This same phenomenon is true in cancer [35,54].

We and others have reported on the dysregulated glucose uptake/metabolism in PAH patients [19,23], animal models [25,55], and primary isolated cell cultures [23,26,28]. In our IPAH 3D culture models, the vascular spheroids had more spontaneous sprouts and increased sprout length compared to controls (Figure 3A,B). When control spheroids were administered PMA (which stimulates glucose uptake through PKC), the control cells increased the vascular sprouts and sprout length at 24 h (Figure 3A,B). In addition, both IPAH PAECs and PASMCs, known to have abnormally high glucose uptake levels and glucose dysfunction [23,28], had increased expression of VEGF ligand at both physiological glucose concentration and higher glucose levels [5 mM (90 mg/dL) vs. 25 mM (450 mg/dL)] compared to control vascular cells (Figure 1A). These data suggest that VEGF-A protein expression may be linked to the increased glucose uptake that has been identified in these cells.

Upon glucose uptake, glucose is rapidly phosphorylated at its carbon-6 position. Then, glucose undergoes many cell fates including metabolism for ATP/energy, pentose phosphate pathway and the hexosamine biosynthetic pathway to generate the sugar nucleotide UDP-GlcNAc, which is fundamental to all glycosylation. In particular, UDP-GlcNAc is a substrate for OGT and ‘GlcNAc’ transfer to many proteins involved in various cell functions including metabolism. Most importantly, OGT has been characterized for its sensitivity to glucose alterations [28,31,35,56]. Multiple reports have shown that OGT/O-GlcNAc is involved in the metastatic, angiogenic, and proliferative processes in cancer cells [35,57,58,59,60,61,62]. Furthermore, some past reports have shown its role in endothelial cells where glucose flux through the HBP and O-GlcNAc regulate the activity of endothelial nitric oxide synthase [38,63,64]. Previously, other reports have documented a connection between O-GlcNAc and angiogenesis in the eye, through SP1/VEGF expression [42], as well as the aorta in diabetic rat models [64,65,66]. Here we show, for the first time, that VEGF ligand expression, which was increased in IPAH vascular cells (Figure 1A), was reduced by OGT knockdown in IPAH PAECs (Figure 1B,C). In addition, blocking OGT activity reduced tube formation and length in IPAH and control PAECs that were shown to be stimulated by VEGF ligand administration (Figure 2). The effect of OGT on vascularization was validated when a reduction in OGT activity led to a decrease in de novo vasculogenesis (Figure 4). These findings suggest that increased OGT/O-GlcNAc levels may regulate the expression of VEGF and contribute to new vessel growth in IPAH vascular cells.

**Figure 5:**
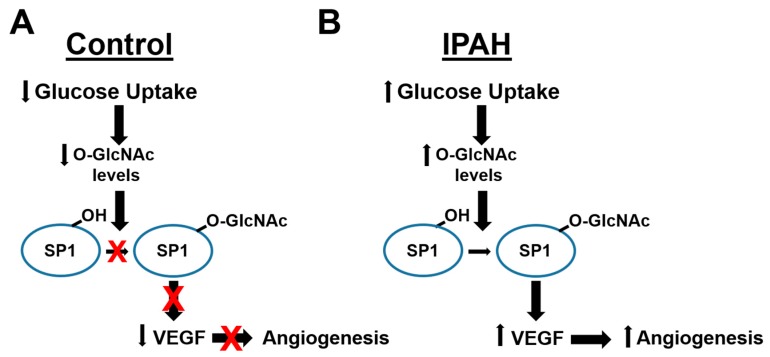
Model of O-GlcNAc regulation of Angiogenesis in IPAH. A schematic illustrating how glucose uptake differences in (**A**) control and (**B**) IPAH vascular cells regulates the O-GlcNAc modification of SP1, VEGF expression, and angiogenesis.

Glucose flux through the HBP has been linked to impaired angiogenesis and vascular dysfunction in many metabolic disorders including diabetes and cancer [30,35,58,59,65,67,68,69,70]. However, the mechanisms have not been completely defined. As stated above, previous reports have shown that OGT can regulate VEGF ligand through the O-GlcNAc modification and activation of SP1. SP1 has been shown to activate/repress many genes following physiological and pathological stimuli and has been shown to be regulated by O-GlcNAc, which indirectly effects other genes [71,72,73,74]. Indeed, SP1 has been shown to modulate genes involved in metabolism and angiogenesis including insulin signaling pathways [74,75]. The increased O-GlcNAc modification of SP1 has been shown to be directly linked to SP1 protein stability [72]. We found that the O-GlcNAc levels of SP1, which could be reduced by OGT inhibition (Figure 1E), were increased in IPAH in high and low glucose concentrations (Figure 1D). In addition, the knockdown of SP1 protein levels led to a decrease in VEGF protein expression in IPAH PAECs (Figure 1F). These data, along with previously published reports, suggest that the increased O-GlcNAc modification of SP1 may contribute to a more stable SP1, which may augment VEGF protein expression and the angiogenic/vasculogenic potential in IPAH vascular cells (Figure 5; Model).

## 4. Materials and Methods

### 4.1. Pasmc and PAEC Cell Culture Conditions

All explanted lungs were collected either at the Cleveland Clinic through an Institutional Review Board approved protocol or they were provided by Baylor, Stanford, Vanderbilt, University of Alabama at Birmingham, and Allegany College of Maryland under the Pulmonary Hypertension Breakthrough Initiative (PHBI). Funding for the PHBI was provided by the Cardiovascular Medical Research and Education Fund (CMREF). Human lung tissues used in this study were from donor lung explants not suitable for lung transplantation and idiopathic PAH patients (Table 1). Human IPAH and control PAECs and PASMCs were isolated from pulmonary arteries dissected from both control and PAH (WHO class IV) lungs obtained at explantation using a previously described method [76]. In some cases, control donor PAECs and PASMCs were obtained from Lonza (Lonza America Inc., NJ, USA). For PAECs, cells were used between passages 5–8 and grown in EBM-2 media with 10% FBS and growth factors (Lonza, Morristown, NJ, USA) unless otherwise specified. Cell purity was confirmed using immunocytochemistry with a panel of endothelial cell-specific markers (23,76). For PASMCs, cells were used between passages 5 and 9 and were grown in low glucose (1 g/L) SMBM-2 media (Lonza, Morristown, NJ, USA) supplemented with growth factors (Growth factor Bullet kit, Lonza, USA) unless otherwise specified. Cell purity was confirmed through positivity staining for α-smooth muscle cell actin, respectively (Sigma-Aldrich, St. Louis, MO, USA). All cells were incubated at 37 °C 5.0% CO_2_ with 90.0% humidity, with media changes at 24 h, followed by a three-day regimen until confluent. For experiments with different glucose concentrations, cells were cultured overnight in Dulbecco’s Modified Eagle Medium (DMEM) glucose-free media (GIBCO, Thermofisher, Waltham, MA, USA) followed by the addition of either 5 or 25 mM glucose (Sigma-Aldrich, St. Louis, MO, USA) for 24 h. Cells were collected for Immunoprecipitation (IP) or Western blot analysis.

### 4.2. Sodium Dodecyl Sulphate-Polyacrylamide Gel Electrophoresis (SDS-PAGE) and Immunoblotting

PASMCs and PAECs were prepared as previously described [26,28] and subjected to SDS-PAGE and Western blot analysis. Nitrocellulose membranes (Bio-Rad, Hercules, CA, USA) were probed with antisera for the following using 5.0% non-fat dry milk in 1 X Tris-buffered saline + tween (TBS-T). Membranes were probed with VEGF (1:5000; Santa Cruz, CA, USA), Sp1 (1:5000; Abcam, Cambridge, MA, USA), OGT (1:5000; clone DM-17, Sigma, MO, USA), or β-actin (1:10,000, Santa Cruz, CA, USA). Following incubation with respective secondary antibodies conjugated with HRP, blots were developed using enhanced chemiluminescence (ECL Prime, Amersham, Pittsburgh, PA, USA) and imaged using a GE Healthcare Imager AI600RGB (GE Healthcare Life Sciences, Pittsburgh, PA, USA). All protein densities were calculated and normalized to β-actin and quantitated using ImageJ [77,78] software.

### 4.3. Immunoprecipitation

IPAH and control primary PAECs were grown to confluency and treated with the addition of either 5 or 25 mM glucose for 24 h (as described above). Cells were then trypsinized and collected for IP. To pre-clear cell supernatants, a 60 μL mixed slurry of unblocked protein L agarose beads (Santa Cruz, CA, USA) was added to 1 g of protein lysate and rotated at 4 °C for 1 h. The agarose beads were removed from pre-cleared cell lysates and antisera against O-GlcNAc-CTD 110.6 antibody (4 ug/1 g protein; Biolegend, San Diego, CA, USA) was added for an overnight IP at 4 °C. O-GlcNAc specific-antibody complexes were purified from lysates using blocked protein L agarose (Santa Cruz Biotech, Dallas, TX, USA) followed by 4 washes using RIPA buffer (Cell Signaling, Danvers, MA, USA), 2 washes with 1.0% Triton-X 100 in 20 mM Tris pH 6.8, and 3 washes with a final wash buffer containing 20 mM Tris-HCl pH 6.8. Partially purified O-GlcNAc modified proteins were eluded from the protein L beads by boiling in 4 X Laemmli buffer (Bio-Rad, Hercules, CA, USA) and subjected to SDS-PAGE. Immunoblot analysis to assess the amount of O-GlcNAc modified SP1 was performed using antisera against SP1 (1:5000, Abcam, Cambridge, MA, USA). In all cases, 5.0% of the whole tissue lysate was set aside as a Western blot input control and probed with SP1 (1:5000, Abcam, MA, USA) and β-actin (as a loading control; 1:10,000, Santa Cruz, CA, USA).

### 4.4. siRNA Knockdown and Cell Culture

Cells were transfected with either human OGT or siRNA (non-target) as previously described (28). Briefly, PASMCs at 70% confluency were transfected with either a scrambled oligonucleotide Silencer^®^ negative control (Thermo Fisher Scientific, Waltham, MA, USA; cat # AM4611), an antisense siRNA oligonucleotide against human OGT (Thermo Fisher Scientific, Waltham, MA, USA; cat # 13301), or against human SP1 (Thermo Fisher Scientific, Waltham, MA, USA; cat # 116546) at 50 nM using HiPerFect transfection reagent (Qiagen, Germantown, MD, USA) and incubated in Opti-MEM (Thermo Fisher Scientific, Waltham, MA, USA) overnight. The following day, transfection medium was changed to the SmGM-2 medium and cells were grown for an additional 48 h and either collected for Immunoblot analysis or subjected to different glucose concentrations (as described above).

### 4.5. PAEC 2-D Tube Formation Assay

To assess PAEC tube formation in control and IPAH cultures, cells were grown using an Angiogenesis assay kit (Millipore, Boston, MA, USA) according to the manufacturer’s protocol. Briefly, uncoated 24-well (MatTek Corp., Ashland, MA, USA) were coated with 150 μL of an ECMatrix solution supplied in the kit and allowed to solidify for 1 h. PAECs were cultured in 10-cm plates, trypsinized, resuspended in medium 199, and seeded at a density of 1.8 × 10^5^ cells/well with either VEGF-A (50 ng/mL), OGT inhibitor (OSMI-1, 25 μM, Sigma-Aldrich, St. Louis, MO, USA), or no treatment and cultured for 6 h. Tube formation was examined using an inverted phase-contrast microscope (Olympus IX71) to assess tube formation at 6 h. Five high-power fields per condition from triplicate experiments were analyzed using Image-Pro^®^ Plus 7.0 (Media Cybernectics, Rockville, MD, USA).

### 4.6. 3-D In Vitro Angiogenesis Model Using PAECs:PASMCs

The 3-D angiogenesis assay was done using a modification of the method described previously [79,80]. Briefly, control and IPAH PAECs and PASMCs cells were cultured initially as a monolayer in their respective media as described above. For generating endothelial and smooth muscle cell co-culture spheroids, cells were trypsinized, collected, and resuspended at a ratio of 4:1 EC:SMC (800:200 cells/spheroid, respectively) into 80% culture medium (50% PAEC medium + 50% PASMC medium) containing 20% Methocel^®^ (Methyl cellulose, Sigma-Aldrich, St. Louis, MO, USA). Cells were then distributed into equally into a 96-well clear round bottom ultra-low attachment microplates (Corning^®^, Corning, NY, USA). Cells were incubated for 24 h to generate co-culture spheroids. For establishing the in vitro angiogenesis assay, freshly generated 4:1 EC:SMC spheroids were embedded into collagen gels. The collagen stock solution was prepared by mixing 1 volume of 10 × Medium 199 (Sigma-Aldrich, St. Louis, MO, USA) to 8 volumes of rat tail collagen solution (2 mg/mL, 4 °C; Corning^®^ Collagen I, Rat Tail, BD Bioscience, San Jose, CA, USA) and immediately inverted to avoid polymerization and placed on ice ~15 min for neutralization. The pH was adjusted to 7.4 by using ~1 volume of 0.1 N NaOH. Spheroids were collected and transferred from the 96-well plate to a conical tube (~50 spheroids/tube) and spun at 200–500× *g* for 3 min. The supernatant was removed and the spheroids were loosened by light agitation and resuspended in a 1:1 mixture of collagen stock solution and room temperature FBS-containing Methocel^®^ (40% FBS + 60% Methocel^®^). At this point, OSMI-1 (25 μM) or PMA (0.5 μg/mL, Sigma, MO, USA) was added to spheroid cultures if required. The spheroid-containing collagen/Methocel^®^ solution was distributed on pre-warmed 24-well (Greiner CELLSTAR^®^ multi-well culture plates, Sigma-Aldrich, St. Louis, MO, USA) plates (each well contains ~30–50 spheroids). Place the plate in incubator at least 15 min to allow collagen polymerization and then add base medium (with inhibitors/activators) to cover gel and continue incubation for 24 h at 37 °C, 5% CO_2_, and 100% humidity. Snapshot images were obtained using an Olympus CKX31 microscope calibrated at the 10 × objective. A minimum of 20 fields and at least 10 spheroids per experimental group were used for the sprouting analysis. Vascular sprouting was quantitated by measuring sprout length using the CellSens Standard digital imaging software (Olympus Corp. Pittsburgh, PA, USA).

### 4.7. De novo Vascularization in a Humanized Angiogenic Mouse Model

The vascularization procedure has been described previously [46,47]. *Generation of cell implants* – Primary human control and IPAH PAECs were grown as described above and plated at a density of 6.67 × 10^6^ cells/mL. A gel matrix solution was prepared on ice in EBM-2 with 25 mM HEPES; 1.5 mg/mL sodium bicarbonate; 10% fetal bovine serum; 1.5 mg/mL rat tail collagen (type I; Corning^®^); and 0.1 mg/mL fibronectin (Calbiochem, Danvers, MA, USA). The pH was adjusted to 7.4 with approximately 5 μL of sterile-filtered 2 N NaOH per mL of solution. For each implant, 0.18 mL cell suspension (1.2 × 10^6^ cells) and 0.42 mL gel matrix solution were combined in a tube, and 0.5 mL was transferred to a 24-well tissue culture plate. The cell/collagen solution was subjected to: 1-one hour at 37 °C and 5% CO_2_ to allow implant to solidify; 2-overlaid with 0.5 mL EGM2; and 3-incubated overnight at 37 °C and 5% CO_2_ with and without OGT inhibitor, OSMI-1 (25 μM). *Implant Surgery*-Twelve week old adult NOD/SCID mice were anesthetized with 2% isoflurane, and the surgical area was prepared with three rounds of alternating 70% isopropanol and 10% betadine washes to prevent infection. A 5 mm incision was made in the lower abdomen and implants containing 1 × 10^6^ IPAH PAECs (soaked in EGM2 media −/+ OSMI-1) were transferred to the incision with the aid of a sterile transfer pipet. The incision was closed using a single 7 mm surgical clip and tissue glue. The surgical procedure was repeated on the opposite side to allow for insertion of the control PAEC implant under the same conditions. Mice were kept warm and monitored during the recovery period. Seven to 14 days after surgery, the implants were harvested along with the adjacent dermis to maintain orientation and prevent curling. The implant was fixed in formalin-free IHC Zinc Fixative (BD Pharmingen) for 24 h and transferred to 70% ethanol indefinitely. Following paraffin embedding, 6 μm sections were cut and stained with anti-human CD31 positive antibody [recognizes human only [46]; clone JC70/A, DAKO, Carpinteria, CA, USA). The animal protocol including pain management, surgical procedure, recovery, and post-operative care was approved by the UAB Institutional Animal Care and Use Committee (APN-20655).

### 4.8. Statistical Analysis

Data were analyzed with Prism 8 (GraphPad Software, Inc., La Jolla, CA, USA) and shown as mean ± SEM using Student’s *t* test and analysis of variance with one-way ANOVA with appropriate post hoc tests for at least three independent experiments. Significance was accepted as a *p*-value less than 0.05.

## 5. Conclusion

Our findings in this report are the first to describe a role for the OGT/O-GlcNAc axis in modulating VEGF expression and vascularization in IPAH. These findings of OGT regulation of de novo vascularization provide greater insight into the potential role that altered glucose uptake and metabolism may have on the angiogenic process and the development of plexiform lesions. The investigation of how increased OGT activity affects molecular processes in metabolic disorders, like IPAH, will open new avenues for vascular research in other pulmonary diseases, including COPD, Asthma, and IPF, which have been associated with widespread metabolic changes and vascular dysfunction [47,49].

## Figures and Tables

**Table 1:** Demographic information for human primary PASMCs and PAECs.

**IPAH PASMCs (*n*)**	**4**
Age, years (MIN–MAX)	41.0 (26–49)
Female	3 (75.0%)
PAH category:	
*Idiopathic PAH*	*3 (75.0%)*
*Heritable PAH (BMPR2 Mutation)*	*1 (25.0%)*
**IPAH PAECs (*n*)**	**4**
Age, years	34.7 (17–49)
Female	4 (100.0%)
PAH category:	
*Idiopathic PAH*	*3 (75.0%)*
*Heritable PAH (BMPR2 Mutation)*	*1 (25.0%)*
**Control PASMCs (*n*)**	**4**
Age, years	53.5 (43–58)
Female	2 (67.0%)
**Control PAECS (*n*)**	**4**
Age, years	45.6 (21–49)
Female	2 (50.0%)

Data presented as mean (range: MAX–MIN) or number (%) as appropriate. PAH = pulmonary arterial hypertension.

## Supplementary Materials

The following are available online at https://www.mdpi.com/1422-0067/20/24/6299/s1.
