# Supplementary material for: O-GlcNAc Transferase Regulates Angiogenesis in Idiopathic Pulmonary Arterial Hypertension
Source: Int J Mol Sci. 2019 Dec 13;20(24):6299. doi: 10.3390/ijms20246299 (PMC6941156; doi:10.3390/ijms20246299)
Supplement: Supplementary file 1 [file ijms-20-06299-s001.pdf]

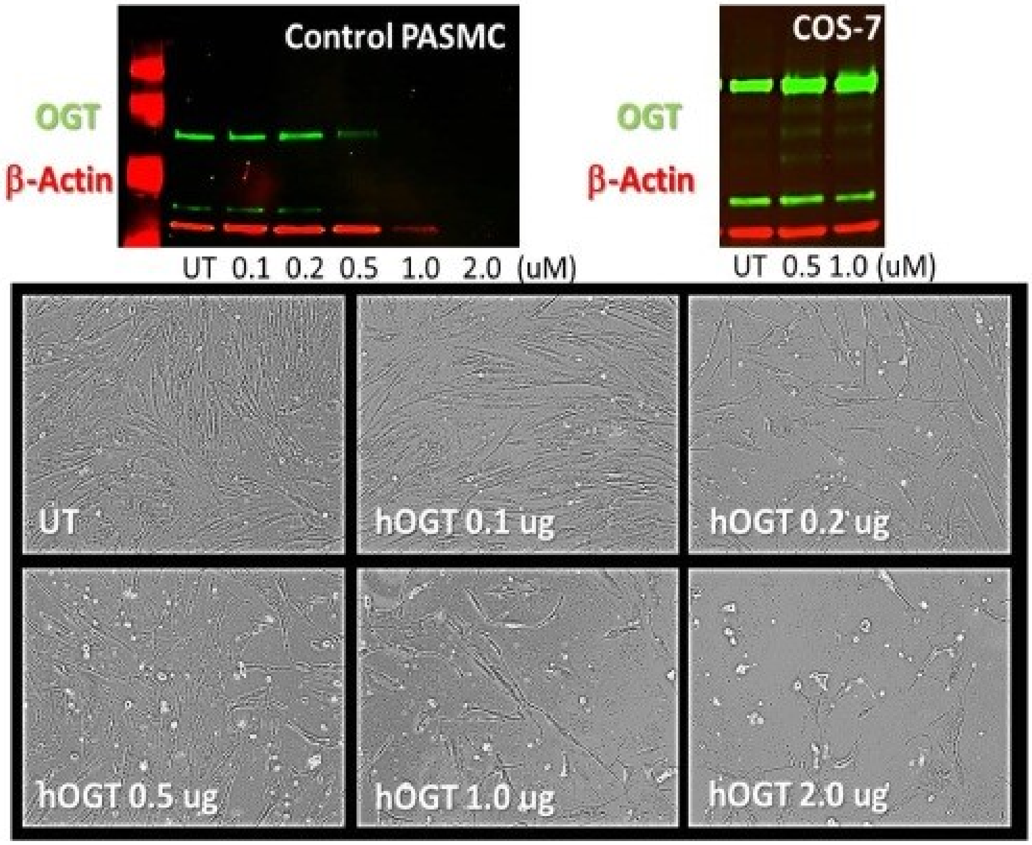

Supplemental figure 1: Dose-dependent cell death in control PASMCs that is not observed in COS-7 cells. PASMCs and COS-7 cells were transfected with different doses of hOGT plasmid. The dose response shown in the control PASMCs shows that the OGT levels do not change at 0.1 and 0.2 compared to the UT. When we increased the amount of plasmid transfected (from 0.5 to 2.0), we observed a dose-dependent cell death response (cell images). The lack of protein loaded reflects the loss of cells due to the overexpression of OGT. All cells were collected at 48 hours for this experiment. Similar findings were observed at a 24hr collection period (data not shown).UT = untransfected, hOGT = human OGT plasmid.
